# Supplementary material for: Effects of the Species and Growth Stage on the Antioxidant and Antifungal Capacities, Polyphenol Contents, and Volatile Profiles of Bamboo Leaves
Source: Foods. 2024 Feb 2;13(3):480. doi: 10.3390/foods13030480 (PMC10855892; doi:10.3390/foods13030480)
Supplement: Supplementary file 1 [file foods-13-00480-s001.zip › foods-2811279-supplementary.pdf]

Table S1. The 30 unidentified VOCs in bamboo leaves.

Compound	CAS#	Formula	MW	RI	RC（%）	CRC（%）
1	unidentified	*	0	1088.8	0.16	
2	unidentified	*	0	1008	0.34	
3	unidentified	*	0	1036.5	0.94	
4	unidentified	*	0	1010.2	0.83	
5	unidentified	*	0	989.7	0.50	
6	unidentified	*	0	1033.5	0.58	
7	unidentified	*	0	1033.9	0.44	
8	unidentified	*	0	1088.4	0.39	
9	unidentified	*	0	870.1	0.12	
10	unidentified	*	0	938.2	1.31	
11	unidentified	*	0	919	0.10	
12	unidentified	*	0	912	0.18	
13	unidentified	*	0	779.1	1.07	
14	unidentified	*	0	781.8	0.82	
15	unidentified	*	0	775.7	0.78	27.9
16	unidentified	*	0	761.4	0.64	
17	unidentified	*	0	787.1	0.35	
18	unidentified	*	0	741.8	1.30	
19	unidentified	*	0	721.4	1.13	
20	unidentified	*	0	717.6	0.79	
21	unidentified	*	0	713.8	0.20	
22	unidentified	*	0	732.4	0.36	
23	unidentified	*	0	741	0.28	
24	unidentified	*	0	740.3	0.63	
25	unidentified	*	0	693.2	3.06	
26	unidentified	*	0	544	1.48	
27	unidentified	*	0	473.6	4.36	
28	unidentified	*	0	440.5	3.29	
29	unidentified	*	0	530.3	1.24	
30	unidentified	*	0	1067.7	0.26	

Note: *, the formula is unknow; MW, Molecular weight; RI, Retention index; RC, Relative composition; CRC, Cumulative relative composition.

Table S2. The relative content of the unidentified compounds in four kinds of bamboo leaves.

Compound	Annual *P. edulis*	Biennial *P. edulis*	Annual *C. delicatus*	Biennial *C. delicatus*
1	0.20 ± 0.02^{b}	0.07 ± 0.01^{d}	0.23 ± 0.01^{a}	0.15 ± 0.00^{c}
2	0.46 ± 0.03^{a}	0.19 ± 0.00^{d}	0.31 ± 0.02^{c}	0.39 ± 0.02^{b}
3	1.18 ± 0.03^{a}	0.81 ± 0.04^{c}	0.79 ± 0.08^{c}	0.98 ± 0.03^{b}
4	0.69 ± 0.05^{b}	0.99 ± 0.05^{a}	0.68 ± 0.06^{b}	0.94 ± 0.01^{a}
5	0.29 ± 0.02^{c}	1.17 ± 0.02^{a}	0.34 ± 0.01^{b}	0.20 ± 0.00^{d}
6	1.36 ± 0.16^{a}	0.15 ± 0.05^{c}	0.39 ± 0.03^{b}	0.39 ± 0.01^{b}
7	0.38 ± 0.08^{c}	0.16 ± 0.07^{d}	0.53 ± 0.06^{b}	0.68 ± 0.02^{a}
8	0.18 ± 0.01^{d}	0.32 ± 0.01^{c}	0.47 ± 0.01^{b}	0.58 ± 0.01^{a}
9	0.22 ± 0.03^{a}	0.18 ± 0.02^{b}	0.04 ± 0.01^{c}	0.05 ± 0.00^{c}
10	0.60 ± 0.03^{c}	0.82 ± 0.07^{b}	1.85 ± 0.10^{a}	1.96 ± 0.01^{a}
11	0.11 ± 0.00^{a}	0.12 ± 0.01^{a}	0.09 ± 0.01^{b}	0.08 ± 0.00^{b}
12	0.10 ± 0.01^{b}	0.19 ± 0.01^{b}	0.34 ± 0.13^{a}	0.09 ± 0.00^{b}

13	1.79 ± 0.20^{a}	1.18 ± 0.07^{b}	0.71 ± 0.03^{c}	0.59 ± 0.01^{c}
14	0.81 ± 0.04^{b}	0.59 ± 0.09^{c}	0.92 ± 0.03^{a}	0.93 ± 0.01^{a}
15	0.71 ± 0.06^{b}	0.65 ± 0.07^{b}	0.88 ± 0.02^{a}	0.86 ± 0.02^{a}
16	0.69 ± 0.04^{b}	0.34 ± 0.03^{c}	0.67 ± 0.03^{b}	0.82 ± 0.01^{a}
17	0.31 ± 0.04^{b}	0.62 ± 0.06^{a}	0.23 ± 0.03^{b}	0.25 ± 0.01^{b}
18	1.86 ± 0.07^{a}	0.79 ± 0.06^{c}	1.24 ± 0.03^{b}	1.29 ± 0.01^{b}
19	0.50 ± 0.00^{d}	1.37 ± 0.03^{b}	1.15 ± 0.04^{c}	1.51 ± 0.02^{a}
20	1.35 ± 0.11^{a}	0.58 ± 0.02^{c}	0.93 ± 0.06^{b}	0.31 ± 0.02^{d}
21	0.22 ± 0.01^{a}	0.22 ± 0.00^{a}	0.19 ± 0.01^{b}	0.19 ± 0.00^{b}
22	0.13 ± 0.02^{c}	0.46 ± 0.02^{a}	0.49 ± 0.04^{a}	0.36 ± 0.01^{b}
23	0.27 ± 0.03^{b}	0.51 ± 0.07^{a}	0.27 ± 0.04^{b}	0.07 ± 0.01^{c}
24	0.69 ± 0.02^{b}	0.83 ± 0.04^{a}	0.54 ± 0.04^{c}	0.47 ± 0.05^{c}
25	3.20 ± 0.40^{b}	1.98 ± 0.10^{c}	2.25 ± 0.11^{c}	4.71 ± 0.08^{a}
26	2.17 ± 0.13^{a}	0.86 ± 0.01^{d}	1.78 ± 0.10^{b}	1.12 ± 0.03^{c}
27	4.47 ± 0.33^{ab}	4.19 ± 0.25^{b}	4.08 ± 0.14^{b}	4.68 ± 0.09^{a}
28	3.92 ± 0.21^{a}	3.74 ± 0.08^{a}	3.40 ± 0.20^{b}	2.15 ± 0.04^{c}
29	1.40 ± 0.05^{a}	1.30 ± 0.01^{b}	1.01 ± 0.05^{c}	1.24 ± 0.01^{b}
30	0.15 ± 0.01^{c}	0.20 ± 0.02^{b}	0.35 ± 0.03^{a}	0.33 ± 0.01^{a}

Data are expressed as means ± SD. The small letter superscripted in each column indicates a sig-nificant difference ($p < 0.05$, n = 3).
